# Anticancer Effects of Bufalin on Human Hepatocellular Carcinoma HepG2 Cells: Roles of Apoptosis and Autophagy

**DOI:** 10.3390/ijms14011370

**Published:** 2013-01-11

**Authors:** Qing Miao, Lin-Lin Bi, Xin Li, Shan Miao, Jin Zhang, Song Zhang, Qian Yang, Yan-Hua Xie, Jian Zhang, Si-Wang Wang

**Affiliations:** 1 Institute of Materia Medica, Fourth Military Medical University, Xi’an 710032, China; E-Mails: miaoqing@fmmu.edu.cn (Q.M.); bilinlin-1979@hotmail.com (L.-L.B.); miaoshan@fmmu.edu.cn (S.M.); yangqianyys@163.com (Q.Y.); xieyanh@fmmu.edu.cn (Y.-H.X.); 2 Department of Pharmacy, Chinese PLA General Hospital, Beijing 100850, China; E-Mail: fmmu_lixin@hotmail.com; 3 Department of Hand Surgery, 401 Military Hospital, Qingdao 266071, China; E-Mail: jinzi1028@126.com; 4 Department of Pharmacy, Tangdu Hospital, Fourth Military Medical University, Xi’an 710032, China; E-Mail: zhangsong801101@163.com; 5 Department of Pulmonary Medicine, Xijing Hospital, Fourth Military Medical University, Xi’an 710032, China

**Keywords:** hepatocellular carcinoma, bufalin, apoptosis, autophagy, AMPK, mTOR

## Abstract

The traditional Chinese medicine bufalin, extracted from toad’s skin, has been demonstrated to exert anticancer activities in various kinds of human cancers. The mechanisms of action lie in its capacity to induce apoptosis, or termed type I programmed cell death (PCD). However, type II PCD, or autophagy, participates in cancer proliferation, progression, and relapse, as well. Recent studies on autophagy seem to be controversial because of the dual roles of autophagy in cancer survival and death. In good agreement with previous studies, we found that 100 nM bufalin induced extensive HepG2 cell apoptosis. However, we also noticed bufalin triggered autophagy and enhanced Beclin-1 expression, LC3-I to LC3-II conversion, as well as decreased p62 expression and mTOR signaling activation in HepG2 cells. Blockage of autophagy by selective inhibitor 3-MA decreased apoptotic ratio in bufalin-treated HepG2 cells, suggesting a proapoptotic role of bufalin-induced autophagy. Furthermore, we investigated the underlying mechanisms of bufalin-induced autophagy. Bufalin treatment dose-dependently promoted AMPK phosphorylation while AMPK inhibition by compound C significantly attenuated bufalin-induced autophagy. Taken together, we report for the first time that bufalin induces HepG2 cells PCD, especially for autophagy, and the mechanism of action is, at least in part, AMPK-mTOR dependent.

## 1. Introduction

Hepatocellular carcinoma (HCC) is the most common type of liver cancer and causes more than 300,000 deaths in China each year [1]. Although surgical resection and liver transplantation have been recognized as the most efficient strategy for HCC treatment [2], most patients failed to meet the criteria because of extensive metastasis and progression. The shortage of healthy donors in conjunction with high medical costs further prompted dropout from the liver transplant waitlist, and has fueled investigation into a wide range of alternative therapies for the management of HCC. HCC is refractory to conventional cytotoxic chemotherapy, adjuvant chemotherapy using doxorubicin, cisplatin, fluorouracil, interferon, epirubicin, or taxol, as single agents or in their combination, however, does not produce a decided survival benefit.

The development and progression of HCC are believed to correlate with dysregulation of cancer cell proliferation and programmed cell death (PCD). Manipulating this balance would therefore provide significant clinical benefits [3]. For instance, sorafenib, a receptor tyrosine kinase inhibitor, has been approved by FDA for patients with advanced HCC [4,5]. The SHARP trial, a large randomized phase III study of patients with biopsy-proven advanced HCC, demonstrated sorafenib improved both overall survival and time to progression compared with the placebo [4]. The mechanism of action lies in sorafenib’s ability to inhibit cancer cell growth and increase apoptosis [6]. To this end, identifying old or new agents with similar pharmacological activities would promote the development of novel therapeutic regimens for HCC.

In recent years, traditional Chinese medicines have attracted increasing interests because of a broad spectrum of biological activities. *Bufo bufo gargarizans* Cantor, *Bufo melanostictus* Schneider, and *Bufo raddei* Sauch are species of toad found to be a source of bufalin, a soluble digoxin-like immunoreactive component also found in Chansu [7]. It has been demonstrated that bufalin exerts cardiotonic, anesthetic, blood pressure stimulation, respiration, and antineoplastic activities [8]. Several preclinical studies indicated bufalin exerts growth inhibition, cell cycle arrest, induces differentiation and apoptosis in gynecologic cancers [9], gastric cancer [10], prostate cancer [11], liver cancer [12], leukemia [13], melanoma [14], lung cancer [15], breast cancer [16], colon cancer [17], and osteosarcoma [18]. The mechanism of bufalin-induced apoptosis has been reported to be the activation of transcription factor AP-1, Rac1, cdc2 kinase, the induction of Tiam1, suppression PI3K/Akt, JNK1/2, ERK1/2 networks, and the elevation of intracellular calcium concentrations. These findings suggest potential clinical benefits of bufalin and drive an increase in ongoing clinical trials [7,19]. Although a few preliminary studies indicate cytotoxic effects of bufalin on HCC [12,20], the exact mechanism of action remains to be defined. Detailed studies for its action on HCC PCD are warranted. We therefore investigate the roles and explore the underlying mechanism of bufalin on PCD in HepG2 cells in the present study.

## 2. Results and Discussion

### 2.1. Bufalin Inhibits HepG2 Cell Proliferation

**Figure 1:**
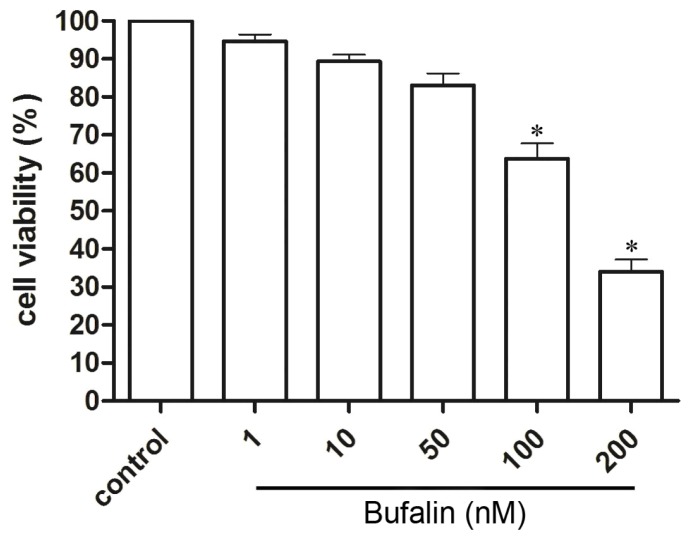
MTT assay of bufalin on HepG2 cells proliferation. Values are expressed as mean ± SD, * *p* < 0.05 *vs.* control.

To access the cytotoxicity of bufalin on HepG2 cells, we performed cell proliferation assay using MTT. HepG2 cells were treated with 1, 10, 50, 100, 200 nM bufalin or vehicle. After incubation for 48 h, cell viability was measured. We found bufalin dose-dependently inhibited HepG2 cells proliferation with an IC_50_ of 143.2 nM (Figure 1). Bufalin at concentrations below 100 nM had minor toxicity for HepG2 cells while bufalin over 100 nM significantly inhibited HepG2 cells proliferation. Therefore, 100 nM bufalin was used in the following experiments.

### 2.2. Bufalin Induces Apoptosis

**Figure 2:**
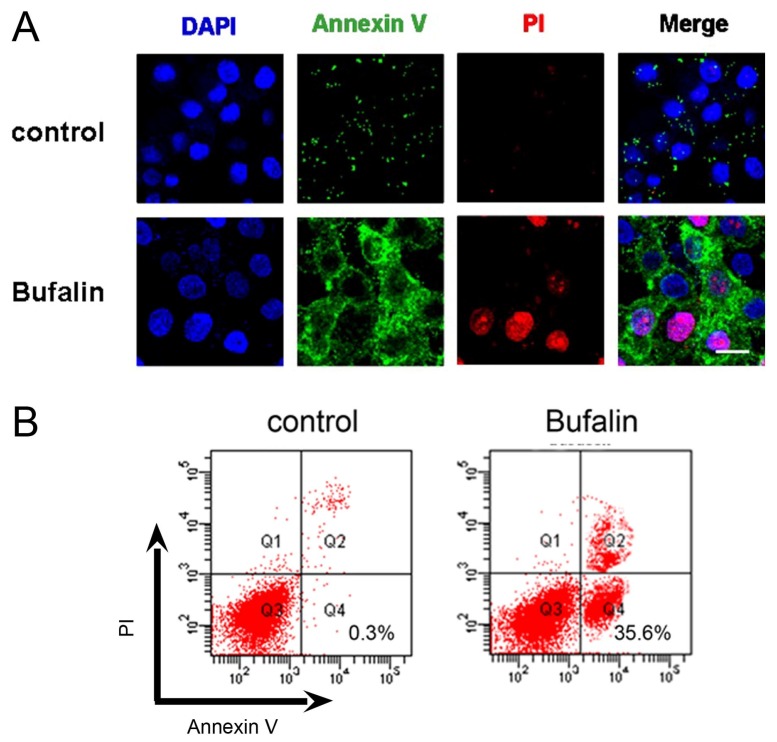
(**A**) Representative images of DAPI, Annexin V-FITC and PI triple fluorescence staining showing HepG2 cell apoptosis after 100 nM bufalin treatment. Cell nucleus was visualized by a blue signal, Annexin V was visualized by a green signal, and PI was visualized by a red signal. Scale bar = 10 μM; (**B**) Flow cytometry analysis of bufalin-induced HepG2 cell apoptosis.

As our previous publications have described that Annexin V-FITC/PI staining suggested cancer cells undergoing apoptosis [21], we next evaluated the effect of bufalin on HepG2 cell apoptosis (also termed type I PCD) using DAPI, Annexin V-FITC and PI triple fluorescence staining. Annexin V-FITC and PI signals could barely be detected in vehicle control cells, while strong fluorescence densities were visible in response to 100 nM bufalin treatment (Figure 2A). We next used flow cytometry to assess the degree of bufalin-induced apoptosis in HepG2 cells. After incubation with 100 nM bufalin for 48 h, bufalin caused ~35.6% population of HepG2 cells apoptosis, which is significantly different from ~0.3% population in the vehicle control group (Figure 2B). These findings are in good consistency with previous studies that bufalin is capable of inducing cancer cell apoptosis, in particular for HCC [12,20].

### 2.3. Bufalin Triggers Autophagy

Despite apoptosis, autophagy also plays crucial roles in cancer cell survival and death, and is gaining increasing interest in cancer research. Autophagy, also termed type II PCD, is a physiologic process that allows sequestration and degradation of the cytoplasmic contents through the lysosomal machinery [22]. Autophagy allows recycling of cellular components and ensures cellular energy supplement during nutrition starvation, infection, and other stress conditions [23]. The hallmark of autophagy lies in the formation of a double membrane organelle known as an autophagosome. The autophagosome subsequently transports to and fuses with lysosome, where the contents are degraded by lysosomal enzyme [24].

**Figure 3:**
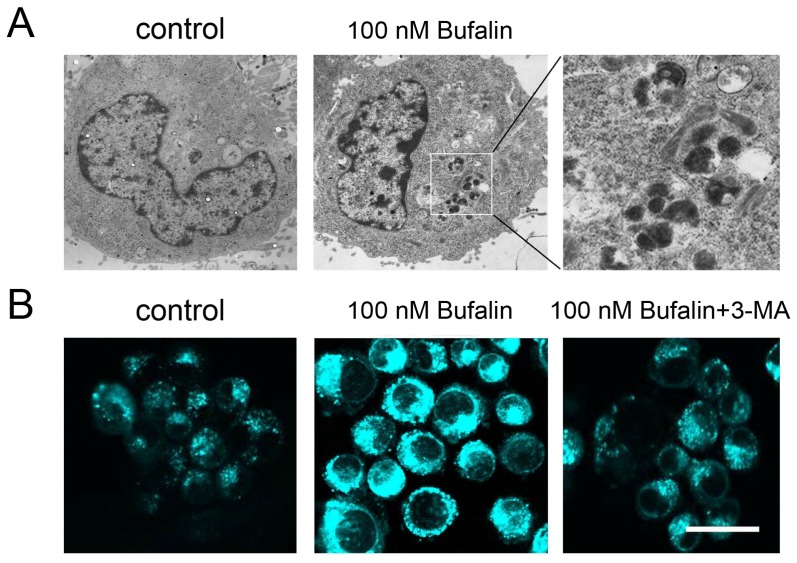
(**A**) Transmission electron microscope image in lower and high power fields of 100 nM bufalin-treated HepG2 cells; (**B**) MDC fluorescent staining of autophagic vesicles in response to 100 nM bufalin without or with autophagic inhibitor 3-MA. Scale bar = 30 μM.

Several lines of studies suggest cytotoxic agents including chemotherapeutic agents induce cancer cell autophagy [25–27]. To date, there is little evidence reporting the autophagic activity of bufalin in cancer cells. In order to shed light on the autophagic profiles of bufalin, we therefore investigated whether bufalin promotes PCD through an autophagy-dependent machinery. Through transmission electron microscopy, no abnormality was detected in the vehicle control HepG2 cells. However, 100 nM bufalin treatment induced inhomogeneous vesicles in the cytoplasma (Figure 3A). At higher power field, these vesicles contained inhomogeneous cytoplasmic contents which were similar to autophagosome-like characters. We also used monodansylcadaverine (MDC) staining to determine if these vesicles were autophagosome. MDC is a fluorescent dye selectively for autophagic vesicles. In non-autophagic cells, MDC fluorescence is diffusely distributed but it exhibits in punctuate vesicular manner when autophagy occurs [28]. Bufalin treatment resulted in accumulation of MDC-labeled vesicles in HepG2 cells. In contrast, pretreatment with 1 mM 3-methyladenine (3-MA, a class III PI3K inhibitor that blocks autophagy) attenuated bufalin-induced MDC fluorescence and autophagic vesicles formation (Figure 3B).

**Figure 4:**
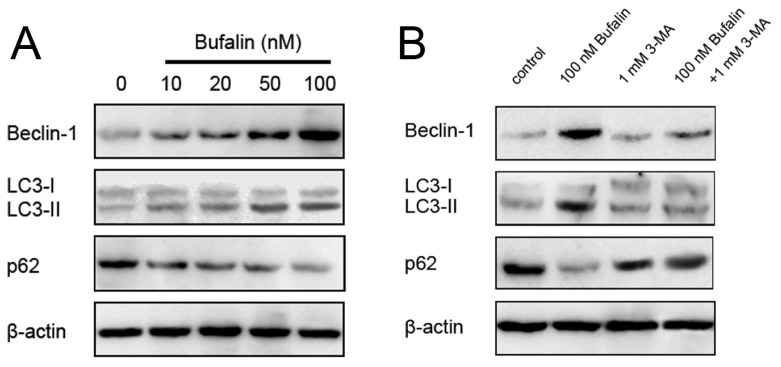
(**A**) Effects of bufalin on the expressions of autophagic indicators Beclin-1, LC3, and p62 in HCC HepG2 cells; (**B**) Autophagy inhibitor 3-MA pretreatment inhibits 100 nM bufalin-induced Beclin-1, LC3 and p62 suppression. β-actin was applied for equal loading.

We next measured expressions of Beclin-1 and microtubule-associated protein-1 light chain-3 (LC3) protein, and p62 (a selective target of autophagy) by western blotting. During autophagy, Beclin-1 was upregulated, LC3-I converted to LC3-II, and p62 was downregulated for the initiation and formation of autophagosomes [29]. The expression of Beclin-1, and LC3-I to LC3-II conversion correlate with the number of autophagosomes and serve as an indicator of autophagy. We observed bufalin dose-dependently upregulated Beclin-1 expression, enhanced LC3-I to LC3-II conversion, and suppressed p62 expression in HepG2 cells (Figure 4A). As expected, 3-MA alone treatment did not affect the expressions of the abovementioned proteins, while combined bufalin and 3-MA treatment markedly inhibited bufalin-induced Beclin-1 upregulation, LC3 conversion, and p62 downregulation (Figure 4B). These results are in agreement with a recent study that bufalin could cause colon cancer cells autophagy [17].

### 2.4. Bufalin-Induced Autophagy as a Proapoptotic Action

**Figure 5:**
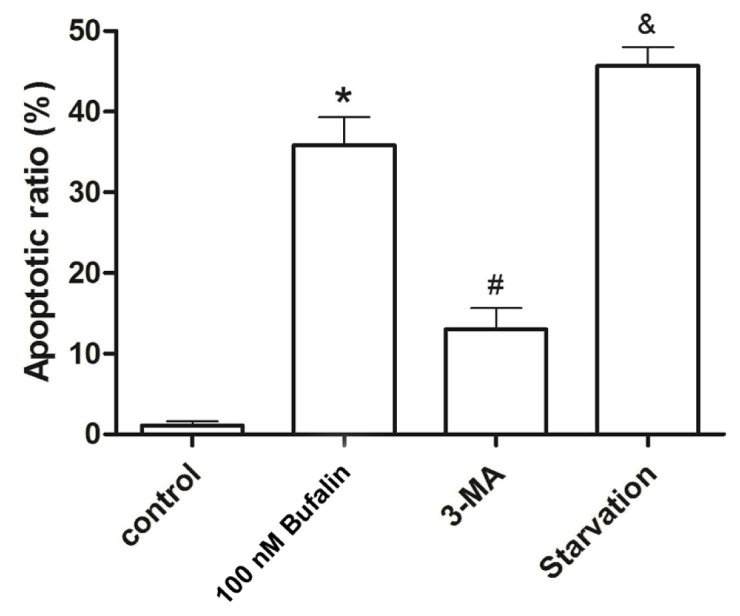
Flow cytometry analysis of HepG2 cells apoptosis in vehicle control, 100 nM bufalin, 3-MA + 100 nM bufalin, and serum starvation + bufalin groups. *p < 0.01 *vs.* control, ^#^*p* < 0.05 *vs.* 100 nM bufalin, ^&^*p* < 0.05 *vs.* 100 nM bufalin.

Because autophagy is initially recognized as an adaptive response to nutrition deficient conditions, and breakdown of autophagosome contents allow non-essential proteins to be degraded and the amino acids recycled for the synthesis of essential proteins [30]. In these regards, autophagy has been considered as a prosurvival action for a long period of time. Nonetheless, emerging explorations suggest prolonged or excessive autophagy would lead to cellular self-degradation followed by apoptosis as well. It is yet unclear whether autophagy contributes to cell death, or rather, represents a prosurvival mechanism. In fact, there is a strong controversy of the exact roles of autophagy in cancer cell treatment. Some experts have declared autophagy as a cytoprotective mechanism while others have argued that autophagic cancer cells ultimately underwent apoptosis [31,32]. To the best of the authors’ knowledge, the current understanding is autophagy plays a dual role in cancer. Autophagy represents prosurvival, as well as proapoptotic mechanisms for cancer cells depending on the cancer type, stage, and microenvironment. As a consequence, both prosurvival and proapoptotic roles are reasonable. However, the molecular mechanisms of this dual role of autophagy are still unclear. Thus autophagy, induced by bufalin, might be used as a therapeutic target only if autophagy can be a proapoptotic action in HepG2 cells. To validate whether bufalin-induced HepG2 cell death attributable to autophagy, we blocked autophagy by 3-MA or induced autophagy by serum starvation. Flow cytometry analysis showed singular 100 nM bufalin treatment caused (35.6 ± 8.4)% apoptosis while 3-MA pretreatment decreased the percentage of apoptosis to (14.2 ± 6.3)%. Interestingly, serum starvation, an effective way to induce extensive autophagy, facilitated bufalin to cause HepG2 cells apoptosis. HepG2 cells cultured in a serum starved condition underwent (44.2 ± 4.8)% apoptosis after 100 nM bufalin treatment, which was significantly different from the non-serum starved condition (Figure 5). These findings strongly suggested bufalin as an inducer of apoptosis and autophagy triggered HepG2 cells PCD. Blockage of autophagy attenuated the process of PCD, but induction of autophagy promoted HepG2 cells apoptosis. Taken together, these results indicate autophagy triggered by bufalin acted as a proapoptotic mechanism, bufalin-induced cell death in HCC cancer cells is dependent, at least in part, on the induction of autophagy.

### 2.5. Bufalin-Induced Autophagy Is AMPK-mTOR Signaling-Dependent

**Figure 6:**
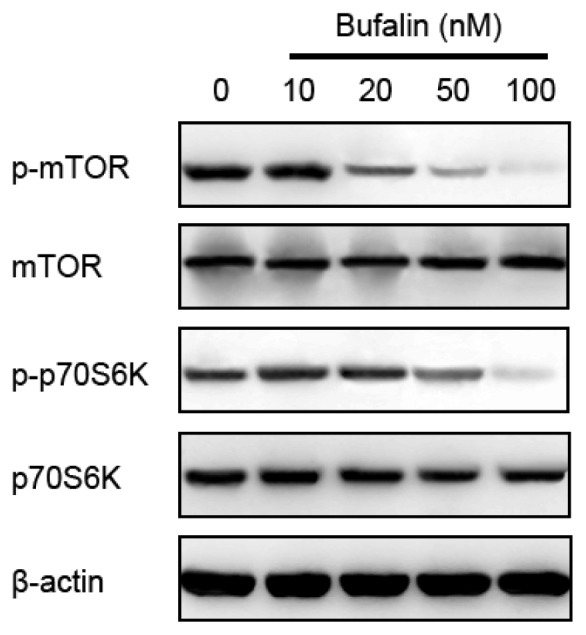
Effects of various doses of bufalin on mTOR signaling detected by Western blot. β-actin was applied for equal loading.

**Figure 7:**
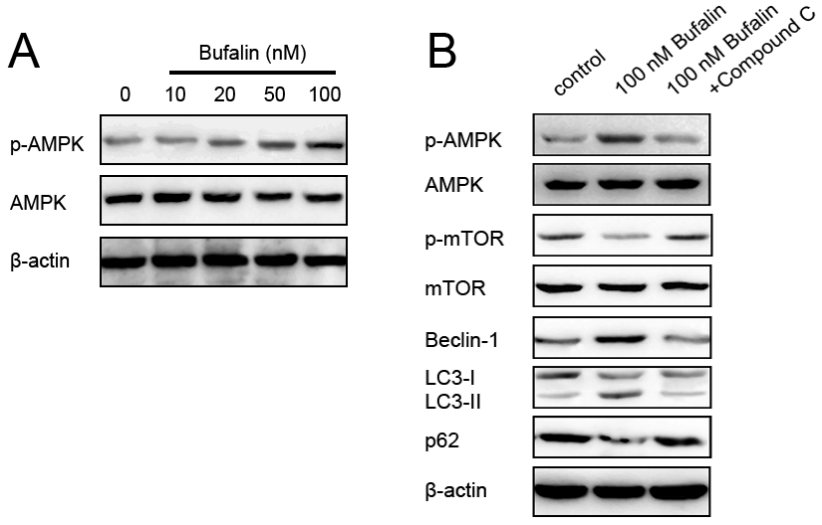
(**A**) Effects of bufalin on the phosphorylation of AMPK in HCC HepG2 cells; (**B**) AMPK inhibitor compound C attenuated 100 nM bufalin-induced mTOR inhibition, Beclin-1 expression, LC3-I to LC3-II conversion, and p62 suppression. β-actin was applied for equal loading.

AMP-activated protein kinase (AMPK) is a principal energy-preserving intracellular enzyme activated in stress conditions that can induce autophagy through inhibition of the serine/threonine kinase mammalian target of rapamycin (mTOR), one major repressor of autophagy [33–35]. The interplay between AMPK-dependent energy metabolism and autophagic adaptive self-sustaining actions has prompted a growing number of investigations in AMPK-mTOR-autophagy mechanisms [36–39]. To elucidate underlying mechanisms responsible for bufalin-induced autophagy in HepG2 cells, we assessed the effects of bufalin on autophagy repressor mTOR and its upstream inhibitor AMPK, respectively. Bufalin treatment of HepG2 cells induced autophagy, which was expectedly followed by decreased phosphorylation of both mTOR and its substrate p70S6K (Figure 6). On the other hand, bufalin dose-dependently induced activation of AMPK (Figure 7A). To this end, we decided to investigate roles of AMPK in bufalin-induced HCC HepG2 cells autophagy. We pretreated HepG2 cells with compound C, a pharmacological inhibitor of AMPK, followed by administration of 100 nM bufalin. According to a recent study, compound C at the concentration of 1 μM was able to inhibit AMPK without cytotoxic effects [40]. We found that 1 μM compound C-mediated inhibition of AMPK activity markedly attenuated bufalin-induced inhibition of mTOR signaling, accumulation of Beclin-1, and conversion from LC3-I to LC3-II (Figure 7B). Thus, the induction of autophagy by bufalin was associated with AMPK activation, mTOR signaling suppression in HCC HepG2 cells.

## 3. Experimental Section

### 3.1. Reagents

**Figure 8:**
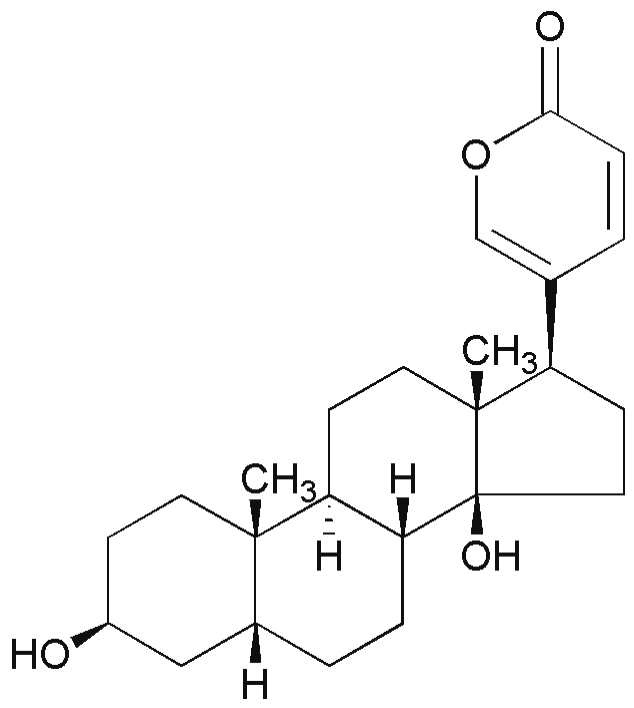
Chemical structure of bufalin, 3-b,14-Dihydroxy-5-beta-bufa-20,22-dienolide.

Bufalin, also termed 3-b, 14-Dihydroxy-5-beta-bufa-20,22-dienolide (Figure 8), was purchased from the Chinese Institute of Pharmacy. Bufalin was dissolved in DMSO and stocked in 1 mM solution in −70 °C. Bufalin was diluted by cell culture medium to indicated concentrations and the final DMSO contents were less than 0.1%.

MTT (methyl thiazolyl tetrazolium), DAPI (diamidino phenyl indole), 3-MA (an autophagy inhibitor), compound C (a pharmacological inhibitor of AMPK) were purchased from Sigma-Aldrich (St. Louis, MO, USA). RPMI-1640 culture medium, and fetal bovine serum were purchased from Gibco (New York, NY, USA). Rabbit anti-Beclin-1, LC3, p-mTOR, mTOR, p70S6K, p-p70S6K, p-AMPK, AMPK, β-actin polyclonal antibodies were purchased from Cell Signal Technology (Danvers, MA, USA). HRP-conjugated goat anti-rabbit IgG were purchased from Abcam (Cambridge, MA, USA). Annexin V/PI apoptotic detection kit was purchased from Kehao Biotechnology (Xi’an, China).

### 3.2. Cell Culture

Human HCC HepG2 cells were purchased from the American Type Culture Collection (ATCC). Cells were routinely cultured in RPMI-1640 medium, supplemented with 10% fetal bovine serum, penicillin (100 U/mL), and streptomycin (100 μg/mL) in an atmosphere with 5% CO_2_ at 37 °C. In all experiments, exponentially growing cells were used.

### 3.3. Cell Proliferation Assay

Cell proliferation was assessed using the MTT assay. Briefly, 5 × 10^3^ cells were incubated in 96-well plates in the presence of various concentrations of bufalin for 48 h. At the end of the treatment, 20 μL MTT (5 mg/mL dissolved in PBS) was added to each well and incubated for an additional 4 h at 37 °C. The purple-blue MTT formazan precipitate was dissolved in 200 μL of DMSO and the optical density was measured at 570 nm.

### 3.4. Measurement of Cell Apoptosis

DAPI, Annexin V, PI triple fluorescent staining for cancer cell apoptosis was performed as we described previously [22]. Briefly, HepG2 cells were cultured in medium containing 100 nM bufalin. After 48 h of treatment, cells were washed twice with 0.01 M PBS and suspended in 200 μL binding buffer. Cells were then incubated with 10 μL DAPI, 10 μL Annexin V-FITC and 5 μL PI for 30 min at 4 °C in dark room. DAPI, Annexin V-FITC and PI fluorescence was immediately observed under confocal laser scanning microscope (Olympus, FV1000, Tokyo, Japan).

For quantitative determination of apoptosis, cells were harvested with trypsinization at the 90% confluent after indicated treatment, stained by Annexin V-FITC and PI as have described, then assessed for cell apoptosis by flow cytometry (Beckman Coulter, CA, USA). Each experiment was performed in triplicate.

### 3.5. Transmission Electron Microscopy of Autophagy

Autophagic HepG2 cells, in response to bufalin treatment, were detected through transmission electron microscopy as we have described [22]. Cell pellets were fixed with 2% glutaraldehyde in 0.1% sodium cacodylate buffer for 12 h at 4 °C. Fixation was followed by 5 min washes with 0.1% sodium cacodylate buffer. Cells were post-fixed with a solution containing 1% osmium tetroxide and 2% K4Fe, stained with 1% uranyl acetate, and pelleted in 2% agar. Pellets were dehydrated in graded ethanol solution and embedded in spur resin. Ultra thin (60 nM) sections were cut on a Reichert Ultra cut microtome, collected on Rhodanimu 400-mesh grids, post-stained with uranyi acetate and lead citrate, and washed with water. The sections were examined in transmission election microscope (Joel, JEM-2000EX, Tokyo, Japan).

### 3.6. MDC Staining

For autophagic fluorescence observation, cells were grown on coverslips and treated with 100 nM bufalin with or without 1 mM 3-MA. After 48 h, cells were stained with 0.05 mM MDC at 37 °C for 1 h. The cellular fluorescence changes were observed using confocal laser scanning microscope (Olympus, FV1000, Tokyo, Japan).

### 3.7. Western Blot

After indicated treatments, cells were collected and washed in ice-cold PBS, and lysed in RIPA buffer (50 mM Tris-HCl pH 7.4, 150 mM NaCl, 1% NP-40, 0.1% SDS, 1 mM PMSF) for extraction of total protein. Protein concentrations were determined and equal amounts of sample were loaded on SDS-PAGE and transferred to nitrocellulose membranes at 400 mA for 1 h. Membranes were stained with 0.5% Ponceau in 1% acetic acid for confirmation. Blots were blocked for 2 h in TBST (10 mM Tris-HCl, pH 7.4, 150 Mm NaCl, 0.05% Tween-20) containing 5% fat-free dried milk and then incubated with the primary antibodies overnight and then incubated with corresponding HRP-labeled secondary antibodies. β-actin was selected as an internal control. Protein bands were visualized by chemiluminescence with the enhanced chemiluminescent detection kit according to the manufacturer’s instructions.

### 3.8. Statistical Analysis

Experiments presented in the figures are representative of three or more different repetitions. Values are expressed as mean ± SD. Clampfit software and ORIGIN6.1 software were used to analyze the data. One-way analysis of variance (ANOVA) was employed to determine the statistical significance between different groups. Significant difference was set at *p* < 0.05.

## 4. Conclusions

Collectively, the present study reports for the first time that bufalin induces apoptosis and autophagy in HCC HepG2 cells. The induction of autophagy by bufalin was associated with the activation of AMPK and the inhibition of mTOR signal pathway. These results suggest that bufalin may be applicable as a medical treatment for HCC. In addition, data from our study indicate that bufalin plays a proapoptotic role and facilitates apoptosis to promote PCD. The mechanisms of action are, at least in part, AMPK-mTOR signaling-dependent.
